# Biomarkers of Renal Disease and Progression in Patients with Diabetes

**DOI:** 10.3390/jcm4051010

**Published:** 2015-05-19

**Authors:** Radovan Hojs, Robert Ekart, Sebastjan Bevc, Nina Hojs

**Affiliations:** 1 Department of Nephrology, Clinic for Internal Medicine, University Clinical Centre Maribor, Ljubljanska 5, Maribor 2000, Slovenia; E-Mails: sebastjanbevc@yahoo.com (S.B.); nina.hojs1@gmail.com (N.H.); 2 Department of Dialysis, Clinic for Internal Medicine, University Clinical Centre Maribor, Ljubljanska 5, Maribor 2000, Slovenia; E-Mail: robert.ekart2@guest.arnes.si; 3 Faculty of Medicine, University of Maribor, Taborska ul. 8, Maribor 2000, Slovenia

**Keywords:** diabetes, nephropathy, biomarkers, tubular biomarkers, inflammation, oxidative stress

## Abstract

Diabetes prevalence is increasing worldwide, mainly due to the increase in type 2 diabetes. Diabetic nephropathy occurs in up to 40% of people with type 1 or type 2 diabetes. It is important to identify patients at risk of diabetic nephropathy and those who will progress to end stage renal disease. In clinical practice, most commonly used markers of renal disease and progression are serum creatinine, estimated glomerular filtration rate and proteinuria or albuminuria. Unfortunately, they are all insensitive. This review summarizes the evidence regarding the prognostic value and benefits of targeting some novel risk markers for development of diabetic nephropathy and its progression. It is focused mainly on tubular biomarkers (neutrophil-gelatinase associated lipocalin, kidney injury molecule 1, liver-fatty acid-binding protein, *N*-acetyl-beta-d-glucosaminidase), markers of inflammation (pro-inflammatory cytokines, tumour necrosis factor-α and tumour necrosis factor-α receptors, adhesion molecules, chemokines) and markers of oxidative stress. Despite the promise of some of these new biomarkers, further large, multicenter prospective studies are still needed before they can be used in everyday clinical practice.

## 1. Introduction

Chronic kidney disease (CKD) is an important public health problem and the prevalence is estimated to be 8%–16% worldwide [1]. Management of CKD is costly and approximately 2 million people are receiving renal replacement therapy [2]. Diabetes prevalence is also increasing rapidly worldwide, mainly due to the dramatic increase in type 2 diabetes [3]. Diabetes is the leading cause of CKD in many developed countries and is also rapidly becoming the leading cause in developing countries as a consequence of the global increase in type 2 diabetes and obesity [2,3]. Diabetic nephropathy occurs in up to 40% of people with type 1 or type 2 diabetes [4]. People with diabetic nephropathy are not only at significant risk of progression to end-stage renal disease (ESRD), there is also a concomitant increase in cardiovascular morbidity and mortality [4]. Hence, it is important to identify patients at risk of diabetic nephropathy and also those at high risk of progression to ESRD. Unfortunately, there is paucity of sensitive and specific biomarker for the early prediction of patients who will develop diabetic nephropathy or will progress to ESRD. Nowadays, in clinical practice, most commonly used markers of renal disease and progression of CKD are estimated glomerular filtration rate (eGFR) and proteinuria. Estimations of GFR reflect late functional changes and not early structural alterations in the kidney. Estimates of declining GFR are also compromised by methodological limitations to estimate GFRs in normal and high range [4,5]. Albuminuria has long been used to monitor onset and progression of diabetic nephropathy [4]. Microalbuminuria was historically considered a strong predictor of progression to proteinuria but recent studies have challenged the predictive value of microalbuminuria, since a spontaneous remission of microalbuminuria has been reported in large proportion of patients with diabetes [4,5,6].

According to the above mentioned, it is clear that new biomarkers are needed to predict patients who will develop diabetic nephropathy or are at risk of progressing to ESRD. Some of the promising new biomarkers in serum and urine will be presented in this paper.

## 2. New Biomarkers of Renal Disease and/or Progression of Renal Disease in Diabetic Patients

### 2.1. Tubular Biomarkers

Tubulointerstitial damage is an important feature of diabetic nephropathy and is, in addition to glomerular damage, an important predictor of renal dysfunction [4,5,6,7]. In the last years, increasing interest in new biomarkers that reflect tubular damage have been noted, among them most studied are neutrophil gelatinase-associated lipocalin (NGAL), kidney injury molecule 1 (KIM-1), and liver-fatty acid-binding protein (L-FABP). Some other biomarkers of tubular damage have also been studied.

### 2.2. Neutrophil Gelatinase-Associated Lipocalin

Neutrophil gelatinase-associated lipocalin (NGAL) is small (25-kDa) protein that is released from injured renal tubular cells in acute kidney injury into the blood and urine, long before a decrease in the glomerular filtration rate can be detected [8,9,10]. Serum and urinary NGAL is an already established biomarker of acute kidney injury (AKI) [11,12]. Elevated levels of serum and urinary NGAL and inverse correlation with GFR have been shown also in CKD patients with autosomal dominant polycystic kidney disease [13]. Elevated levels of urinary NGAL have been shown in primary focal segmental glomerulosclerosis [14] and urinary NGAL has also been shown to predict worsening of kidney function in membranous nephropathy [15]. Urinary NGAL levels also correlated with the degree of proteinuria in patients with IgA nephropathy [16] and membranous nephropathy [14,15].

In one of the first large studies, Bolignano *et al.*, included 96 patients with CKD stages 2–4, among them 20% with diabetic nephropathy [17]. Patients were followed prospectively until the end of the observation period (20 months) or the primary study endpoint, defined as doubling of baseline serum creatinine and/or the onset of ESRD, was reached. Baseline urine and serum NGAL levels predicted CKD progression in univariate and multivariate analysis independently of other potential confounders, including eGFR and age. In the study by Yang *et al.*, 74 type 2 diabetic patients were included [18]. They were divided into normo-, micro- and macro-albuminuria groups according to their 24-h urinary albumin excreting rate and were followed for one year. Average baseline serum NGAL values decreased from normo-albuminuria group to macro-albuminuria group and increased in opposite way for urine NGAL. The same results have been found after one year of follow up. Despite the lack of an adequate explanation, authors suggested that serum NGAL may increase in the very early stage of diabetic nephropathy and drop down as the disease develops; as the disease progresses, excretion of NGAL in urine increases and the absorption function of tubule decreases. In the large study by Smith *et al.*, 158 patients with CKD stage 3 and 4 were included, and 6% were patients with diabetic nephropathy [19]. The baseline urine NGAL-to-creatinine ratio was associated with initiation of renal replacement therapy independent of conventional renal risk factors, including proteinuria. Higher baseline urine NGAL-to-creatinine ratio also independently predicted rapid renal decline (≥5 mL/min/1.73 m^2^) over one year. Adding urine NGAL-to-creatinine ratio demonstrated the greatest benefit in identifying patients with CKD progression only in group of patients with relatively low proteinuria. In the study by Nauta *et al.*, 94 diabetic (41 normoalbuminuric, 41 microalbuminuric, 12 macroalbuminuric) and 45 non-diabetic control subjects were included [20]. Urinary NGAL was significantly elevated in normoalbuminuric diabetic patients compared with nondiabetic control subjects and concentration increased per albuminuria stratum. Urinary NGAL was significantly associated with the eGFR in univariate model but not after adjusting for age, sex, and albuminuria. Similar results were shown in the study by Nielsen *et al.*, in 63 type 1 diabetic patients with diabetic nephropathy who were followed for three years [21]. According to urine NGAL, patients were divided into quartiles and those in highest quartile had significant decrease in GFR each year. In a linear regression analysis, high levels of urine NGAL were associated with a faster decline in GFR but after adjustment for other known disease progression markers, among them also proteinuria, the association was not significant anymore. In the Taiwan study by Chou *et al.*, serum and urine NGAL were used in addition to albuminuria to predict the GFR decline rate in type 2 diabetic patients, but only urine albumin excretion rate was significantly associated with eGFR and the eGFR decline rate [22].

According to conflicting results of previously mentioned studies, and due to the strong link with proteinuria in most of the studies, serum and urine NGAL do not offer additional prognostic information compared to already established biomarkers.

### 2.3. Kidney Injury Molecule 1

Kidney injury molecule 1 (KIM-1) is a transmembrane tubular protein with unknown function, not detectable in the normal kidney, but elevated in experimental and clinical kidney damage [23]. It is a phosphatidylserine receptor and it seems that it plays an important role in apoptosis by recognizing apoptotic cells and directing them to lysosomes [24]. It also serves as a receptor for oxidized lipoproteins and hence is adept at recognizing apoptotic cell “eat me” signals [24]. It was shown that it is increased in the urine in AKI [25]. It was also shown that it is a promising biomarker for early detection of AKI with considerable predictive value in a recently published meta-analysis [26]. Experimental studies suggest that KIM-1 may be an indicator of AKI to CKD transition [27].

In the study by von Timmeren *et al.*, in biopsies from various renal diseases (102 patients, eight with diabetic nephropathy) and controls, renal KIM-1 was significantly increased in all diseases *versus* controls, except minimal change [23]. KIM-1 was primarily expressed at the luminal side of dedifferentiated proximal tubules, in areas with fibrosis and inflammation [23]. Renal KIM-1 correlated positively with renal damage, negatively with renal function, but not with proteinuria [23]. At the time of biopsy in 53 patients and in 11 healthy subjects, urinary KIM-1 was measured [23]. Urinary KIM-1 was increased in renal patients *versus* controls and correlated positively with tissue KIM-1 and negatively with renal function, but again not with proteinuria [23]. In the prospective study by von Timmeren *et al.*, 145 renal transplant recipients were included and were followed for four years for graft loss [28]. At baseline, 24 hour urine samples were collected for assessment of urinary KIM-1. Occurrence of graft loss increased over tertiles of KIM-1 excretion, high KIM-1 excretion was associated with proteinuria, low creatinine clearance, and high donor age [28]. In multivariate analyses, prediction of graft loss by KIM-1 was independent of creatinine clearance, proteinuria, and donor age [28]. Urinary KIM-1 concentrations were associated with a faster decline in GFR also in the two large studies including type 1 or type 2 diabetic patients, but in both studies, after adjustment for known promoters of progression of renal disease, KIM-1 concentrations were not independently associated with a faster decline in GFR [21,22,23,24,25,26,27,28,29].

### 2.4. Liver-Fatty Acid-Binding Protein

Liver-fatty acid-binding protein (L-FABP) is an intracellular carrier protein that is expressed in proximal tubules of the kidney and liver. Although its precise function is unknown, it is believed to be endowed with protective functions [30]. L-FABP in the kidney has been postulated to represent an endogenous anti-oxidant capable of suppressing tubulointerstitial damage [31]. L-FABP is emerging as excellent biomarker for the early prediction of AKI [32]. Urinary excretion of L-FABP is also increased in CKD and is associated with structural and functional tubular damage [33]. In the study including 120 nondiabetic CKD patients, urinary L-FABP levels correlated with proteinuria and serum creatinine and were associated with progression of CKD [34].

In the large prospective study by Nielsen *et al.*, including 165 normoalbuminuric type 1 diabetic patients urinary L-FABP predicted the development of micro- and macro-albuminuria independent of recognized biomarkers [35]. In another large study by Kamijo-Ikemori *et al.*, including over 100 type 2 diabetic patients, high urinary L-FABP levels were associated with the increase in albuminuria, progression of diabetic nephropathy to ESRD or induction of haemodialysis [36]. In the last large study by Chou *et al.*, only urine albumin excretion rate was significantly associated with eGFR and eGFR decline rate in type 2 diabetic patients and not L-FABP [29].

In the study by Nauta *et al.*, including type 2 diabetic patients, heart fatty acid-binding protein (H-FABP), a marker of distal tubular damage, was the only tubular marker associated with eGFR after adjustment for other risk markers of progressive diabetic nephropathy [27].

According to published studies, members of the FABP family are emerging as the tubular markers with the greatest chance of offering added predictive value for progressive diabetic nephropathy over and above that offered by established risk markers [4].

### 2.5. Urinary N-Acetyl-Beta-d-Glucosaminidase

Urinary *N*-acetyl-beta-d-glucosaminidase (NAG) is a lysosomal enzyme that is constitutively expressed by the proximal tubule and a well-studied urinary marker of established proximal tubule cell injury [37]. NAG is elevated in the urine in patients with glomerulonephritis compared with healthy controls [38].

In the study by Vaidya *et al.*, lower urinary NAG levels were associated with the regression of microalbuminuria in type 1 diabetes mellitus [39]. In the large study by Kern *et al.*, including type 1 diabetic patients who participated in the Diabetes Control and Complications Trial (DCCT), baseline levels of urinary NAG independently predicted the development of micro- and macro-albuminuria during the follow up period of nine years [40]. The lack of data about the use of ACE inhibitors in this study is an important limitation. In addition, several studies have demonstrated a direct correlation between NAG excretion and hyperglycaemia; this confounding variable may further limit the utility of NAG as a biomarker in diabetic subjects [41]. Further studies are required to clarify the role of NAG as a biomarker in diabetic nephropathy.

## 3. Markers of Inflammation and Oxidative Stress

Several factors are involved in the development and progression of diabetic nephropathy. Growing evidence indicates that pathogenesis and progression of diabetic nephropathy is associated with the presence of a chronic subclinical low-grade inflammatory state and oxidative stress [42,43]. Increases in oxidative stress can increase the production of inflammatory cytokines and, likewise, an increase in inflammatory cytokines can stimulate the production of free radicals [43].

## 4. Inflammation

### 4.1. Pro-Inflammatory Cytokines

Cytokines are low molecular weight polypeptides and their most important function is regulation of the inflammatory process. They contribute in accelerating and maintaining chronic inflammation. The first studies suggesting the role of inflammatory cytokines in the development of diabetic nephropathy were published more than 20 years ago [44,45].

Interleukin-6 (IL-6) is a proinflammatory cytokine, which is produced by many cells (adipocytes, activated leucocytes, myocytes and endothelial cells) and is also associated with visceral obesity and insulin resistance [46]. In the study by Saraheimo *et al.*, 194 type 1 diabetic patients from multi-centre Finnish Diabetic Nephropathy Study (FinnDiane) were included [47]. They were divided into three groups according to their albumin excretion rate (normo-, micro- and macro-albuminuria) [47]. In this study, IL-6 increased in parallel with the severity of the renal disease (albuminuria) and in multiple regression analysis albumin excretion rate, HDL-cholesterol and duration of diabetes were independently associated with IL-6 [47]. In the study by Tong *et al.*, including 29% of patients with diabetic nephropathy baseline IL-6 levels gradual increase from control subjects to CKD stage 3 and 4 and to CKD stage 5 patients [48]. In the study by Wolkow *et al.*, five inflammatory markers—IL-6, IL-8, monocyte chemoattractant protein 1 (MCP-1), interferon γ-inducible protein (IP-10), and macrophage inflammatory protein 1d—were measured in urine samples collected from individuals with type 1 diabetes [49]. Baseline urinary concentrations of these inflammatory markers were found to be significantly higher in those with an early progressive kidney function decline compared with those who displayed stable kidney function [49]. By multivariate analysis, those with more than two elevated marker levels were found to be more than five times as likely to have an early progressive decline in kidney function [49].

In the study by Moriwaki *et al.*, 151 type 2 diabetic patients with various degrees of nephropathy, as well as 80 healthy volunteers, were included [50]. Significant differences in serum levels of IL-18 were observed between the patients and control subjects, whereas that of IL-6 was not different between the two groups, although patients with nephropathy showed higher levels [50]. In addition, IL-6 and IL-18 levels were increased in diabetic patients with microalbuminuria or clinical albuminuria compared with those without albuminuria [50].

In the another study by Nakamura *et al.*, serum and urinary IL-18 and serum IL-6 levels were also significantly elevated in patients with type 2 diabetes compared to control subjects [51]. In uni- and multi-variate analyses, albuminuria excretion ratio was independently associated with serum and urinary IL-18 levels. Moreover, serum and urinary IL-18 levels correlated positively with albuminuria after 6 months and changes in albuminuria during the follow-up period [51].

### 4.2. Tumour Necrosis Factor-α and Tumour Necrosis Factor-α Receptors

The primary source of tumour necrosis factor-α (TNF-α) are monocytes and macrophages, although intrinsic renal cells are also able to synthesize this cytokine [52]. It was shown in an experimental model that renal mRNA expression of TNF-α is significantly increased in diabetic rats compared with normal rats [53,54]. Navarro *et al.*, reported in two studies direct and significant association between serum TNF-α and urinary protein excretion in diabetic patients with normal renal function and microalbuminuria, as well as in subjects with overt nephropathy and renal insufficiency [55,56]. Urinary TNF-α levels were also elevated in diabetic patients with increased urinary albumin excretion and there was a significant rise in urinary TNF-α excretion as diabetic nephropathy progressed [55,56]. Multivariate analysis shows a significant and independent relationship between urinary TNF-α and urinary albumin excretion. In these studies, a significant correlation between serum and urinary concentrations was not found, suggesting an intrarenal production of TNF-α [55,56]. Similar results were found by Moriwaki *et al.*, where significant differences in serum levels of TNF-α were observed between the type 2 diabetic patients and control subjects [50]. In patients with microalbuminuria or clinical albuminuria, TNF-α levels were significantly increased compared to those without albuminuria [50].

Interestingly, in the study by Niewczas *et al.*, only levels of soluble forms of receptors 1 and 2 for TNF-α, and not TNF-α, were found to be associated with GFR in a multivariate analysis [57]. In another study by Niewczas *et al.*, 410 patients with type 2 diabetes were included and were followed for 12 years [58]. In follow-up period, 59 patients developed ESRD. Of the examined markers, only TNF receptors 1 and 2 were associated with risk for ESRD and TNF receptor 1 predicted risk for ESRD even after adjustment for clinical covariates such as urinary albumin excretion [58]. In the study by Gohda *et al.*, 628 patients with type 1 diabetes, normal renal function and no proteinuria were included and followed for 12 years; in the follow up period, 69 patients developed eGFR less than 60 mL/min per 1.73 m^2^ [59]. In this study, early GFR loss was associated with circulating TNF receptor 1 and 2 levels but not TNF-α levels [59]. Similar results were reported in the study based on DCCT including type 1 diabetic patients [60]. Circulating TNF receptors 1 and 2 were important independent predictors of the development of macroalbuminuria [60]. Despite these studies suggesting that serum TNF receptor levels are associated with the development and progression of diabetic nephropathy, the exact mechanisms linking TNF receptors with diabetic nephropathy is not known and further studies are needed.

### 4.3. Adhesion Molecules

Infiltration of leukocytes into inflammatory lesions is mediated by adhesion to endothelial cells and transmigration from vascular lumen to inflammatory sites [61]. Adhesion molecules are expressed on the cell surface, and mediate cell-cell binding and cell-matrix attachment [61]. Leukocyte adhesion to vascular endothelial cells is promoted by adhesion molecules expressed on leukocytes and endothelial cells. Selectin molecules mediate the leukocyte rolling along with endothelial cells at the first step of leukocyte infiltration into inflammatory lesions. At the second step, tight adhesion of leukocytes to the endothelium is mediated by intercellular adhesion molecule-1 (ICAM-1) and vascular cell adhesion molecule-1 (VCAM-1) [61,62].

In the study by Clausen *et al.*, type 1 diabetic patients and healthy subjects were included [63]. In this study, plasma concentration of ICAM-1 was elevated in microalbuminuric patients and in patients with overt nephropathy, plasma VCAM-1 was elevated only in patients with overt nephropathy [63]. In the study by Lin *et al.*, baseline levels of ICAM-1 and VCAM-1 were measured from stored blood samples from the 1441 participants of the DCCT [64]. Only an elevated baseline plasma level of ICAM-1 was associated with an increasing rate of urinary albumin excretion and the onset of microalbuminuria [64]. In the study by Rubio-Guerra *et al.*, 30 hypertensive type 2 diabetic patients and 30 non-diabetic normotensive subjects were included; their VCAM-1, ICAM-1 and selectin levels were measured [65]. The diabetic patients had significantly higher levels of all circulating soluble adhesion molecules than control subjects and significant correlation only between VCAM-1 levels and 24 h urinary albumin excretion were found [65]. Due to the conflicting results of these studies, further research in this field is required.

### 4.4. Chemokines

Growing evidence suggests that recruitment of inflammatory cells from the circulation into renal tissue plays a pivotal role in the progression of various renal diseases, including diabetic nephropathy. Infiltration of activated T cells and monocytes initiate renal damage and lead to a progressive loss of renal function [66,67]. Chemokine (CC motif) ligand 2 (CCL2) is a small cytokine belonging to the CC chemotactic chemokine family that is also known as monocyte chemotactic protein-1 (MCP-1) [43]. Experimental studies have demonstrated that MCP-1-mediated macrophage accumulation and activation is a critical mechanism in the development of early diabetic nephropathy [68].

In the study by Wada *et al.*, 45 type 2 diabetic patients and 20 healthy subjects were included [69]. Urinary MCP-1 levels were elevated in patients with diabetic nephropathy as compared with those of healthy subjects. In contrast, serum levels of MCP-1 in diabetic nephropathy remained similar to those of healthy volunteers. Urinary levels of MCP-1 were low in patients without protein excretion and slightly, but not significantly, elevated in patients with microalbuminuria. Only in patients with massive proteinuria (nephrotic syndrome) significantly elevated urinary MCP-1 levels were found. In another study by Tashiro *et al.*, levels of urinary MCP-1 in type 2 diabetic patients with normal renal function were significantly higher than those in healthy adults, and urinary MCP-1 levels increased gradually from normo-, micro- to macro-albuminuria [70]. Similar results were found in the study by Morii *et al.*, urinary levels of MCP-1 in patients with macroalbuminuria were significantly elevated compared to the levels in patients with normo- and micro-albuminuria [71]. In this study including type 2 diabetic patients, urinary MCP-1 levels positively correlated with urinary excretion levels of albumin in all subjects [71]. In previously mentioned study by Wolkow *et al.*, in type 1 diabetic patients, baseline urinary MCP-1 was significantly higher in those with an early progressive kidney function decline compared with those who displayed stable kidney function [49].

Two interesting studies demonstrating the effect of treatment with ACE inhibitors and spironolactone on MCP-1 in type 2 diabetic patients have been published. In the first, Amann *et al.*, showed that blockade of the renin–angiotensin system in patients with type 2 diabetes was associated with a reduction in urinary MCP-1 levels as well as an improvement in renal function [72]. In the second, Takebayashi *et al*., found that aldosterone blockade by spironolactone may offer beneficial renoprotective effects through anti-inflammatory mechanisms via the modulation of MCP-1 [73].

## 5. Oxidative Stress

Under normal physiological conditions, there is a balance in the generation of oxygen-free radicals and the antioxidant defence mechanisms used to deactivate free radical toxicity [43]. Experimental studies have highlighted the importance of oxidative stress, in particular the generation of reactive oxygen species (ROS), in type 1 and type 2 diabetes mellitus, triggered in large part by hyperglycaemia [74]. Clinically, patients with diabetes have increased plasma levels of ROS biomarkers such as 8α isoprostanes, oxidised LDL, *etc.*, and reduced levels of antioxidant agents and enzymes, such as bilirubin, superoxide dismutase and antioxidant vitamins, thus promoting oxidative stress [75,76]. Measurement of urinary 8-oxo-7,8-dihydro-2′-deoxyguanosine (8-OHdG) is frequently used to assess oxidative stress in the human body [77,78]. 8-OHdG is a product of oxidative DNA damage following specific enzymatic cleavage after ROS induced 8-hydroxylation of the guanine base in mitochondrial and nuclear DNA [77,78]. Urinary 8-OHdG is directly related to the DNA oxidation ratio and effectiveness of DNA repair [77,78].

In the large study by Hinokio *et al.*, 396 Japanese type 2 diabetic patients with normoalbuminuria or microalbuminuria were included and followed for five years [77]. Authors found a significant progression of diabetic nephropathy in the patients with higher excretion of 8-OHdG in urine compared with the patients with moderate or lower excretion of 8-OHdG.

The multivariate logistic regression analysis suggested that the urinary 8-OHdG was the strongest predictor of nephropathy among several known risk factors. In the another study by Serdar *et al.*, 52 patients with type 2 diabetes mellitus (32 with nephropathy and 20 without) and 20 healthy control subjects were included [78]. The concentrations of urine 8-OHdG were higher in diabetic patients than those of the control subjects. No statistical difference was found between diabetic patients with or without nephropathy. Urinary 8-OHdG did not improve the ability to determine which patients are at risk of progressive diabetic nephropathy over and above measuring urinary albumin to creatinine ratio.

According to conflicting results of clinical studies, it is questionable if markers of oxidative stress add additional prognostic information in relation to the development or progression of diabetic nephropathy compared to established risk markers.

## 6. Conclusions

Today, in clinical practice, most commonly used markers of renal disease and progression of CKD and also diabetic nephropathy are serum creatinine, eGFR and proteinuria/albuminuria but unfortunately they are all insensitive. Some new biomarkers reviewed in this paper are promising but further large, multicenter prospective studies are needed before they can be used in everyday clinical practice. The main problem is that most of the biomarkers are still at an intermediate phenotype level, which is too distant from the gene level and most of these biomarkers are deeply influenced by environment, genetics, sex differences, *etc.* [79]. It is also important to determine whether these newly identified biomarkers are purely associations or real biomarkers of underlying pathophysiological processes.

Despite that the purpose of this paper is to present the biomarkers of renal disease and progression in patients with diabetes, it is also important and should be mentioned that, in patients with diabetic nephropathy, there is also a concomitant increase in cardiovascular morbidity and mortality [4]. Some of the previously presented biomarkers are not only biomarkers of renal disease and progression but are also the biomarkers of cardiovascular disease like L-FABP which has been related to cardiovascular morbidity/mortality in type 1 and type 2 diabetic patients [35,36,37,38,39,40,41,42,43,44,45,46,47,48,49,50,51,52,53,54,55,56,57,58,59,60,61,62,63,64,65,66,67,68,69,70,71,72,73,74,75,76,77,78,79,80].
